# Polyaniline photoluminescence quenching induced by single-walled carbon nanotubes enriched in metallic and semiconducting tubes

**DOI:** 10.1038/s41598-018-27769-4

**Published:** 2018-06-22

**Authors:** Mihaela Baibarac, Adelina Matea, Monica Daescu, Ionel Mercioniu, Sophie Quillard, Jean-Yves Mevellec, Serge Lefrant

**Affiliations:** 1 National Institute of Materials Physics, Laboratory of Optical Processes in Nanostructured Materials, P.O. Box MG-7, R077125, Bucharest, Romania; 2 Faculty of Physics, University of Bucharest, 405A Atomistilor, P.O. Box MG-1, 077125 Bucharest, Romania; 3 Institut des Mateŕiaux “Jean Rouxel”, 2 rue de la Houssinieŕe, B.P. 32229, F-44322 Nantes, cedex 3 France

## Abstract

The influence of single-walled carbon nanotubes enriched in semiconductor (S-SWNTs) and metallic (M-SWNTs) tubes on the photoluminescence (PL) of polyaniline (PANI), electrosynthesized in the presence of the H_2_SO_4_ and HCl solutions, is reported. The emission bands peaked at 407–418 and 440–520 nm indicate that the electropolymerization of aniline (ANI) leads to the formation of short and longer macromolecular chains (MCs), respectively. We demonstrate that the reaction product consists of ANI tetramers (TT) and trimers (TR) as well as PANI-salt. Using Raman scattering and IR absorption spectroscopy, a covalent functionalization of SWNTs with shorter and longer MCs of PANI-salt is demonstrated. The presence of S-SWNTs and M-SWNTs induces a decrease in ANI TT weight in the reaction product mass consisting in S-SWNTs and M-SWNTs covalently functionalized with PANI-emeraldine salt (ES) and PANI-leucoemeraldine salt (LS), respectively. A PANI PL quenching is reported to be induced of the S-SWNTs and M-SWNTs. A de-excitation mechanism is proposed to explain PANI PL quenching.

## Introduction

In the last thirty years, many efforts have been made in order to understand the effect of various electrolytes such H_2_SO_4_ and HCl on the polyaniline (PANI) electrosynthesis, the electrochemical properties and the conductivity^1–5^. In this context, the electrochemical generation of PANI was reported to be dependent of anion type^1–3^. The growth of the charge as increasing the cyclic voltammograms number was reported to decrease in the order HSO_4_^−^ > Cl^−^ > ClO_4_^−^ as a consequence of the various nucleation mechanisms of PANI onto the Pt electrode surface^1–3^. A higher conductivity of the PANI doped HSO_4_^−^ ions fibers was determined compared to that reported in the case of the PANI doped with Cl^−^ ions fibers^1^. The electrochemical synthesis was also used to obtain composites based on PANI and carbon nanotubes^6^. Despite a sustained effort in this direction, studies carried out using composites based on PANI and single-walled carbon nanotubes (SWNTs), synthetized by chemical ways^7^ or electrochemical methods^6^, were performed on samples consisting of mixtures of 33% metallic tubes and 66% semiconducting tubes. According to our early studies, it was demonstrated that the oxido-reduction processes of SWNTs films in the presence of (i) H_2_SO_4_ induces both a doping of carbon nanotubes with bisulfate anions and a breaking of SWNTs when carbon nanotubes fragments of different lengths were obtained^8^ and (ii) HCl leads only to a doping of SWNTs with Cl^−^ ions, at potentials smaller than +1000 mV vs. SCE^9^. Taking into account all these and in order to highlight the influence of the SWNTs highly separated in metallic tubes (98%) and semiconducting tubes (99%) on the aniline electropolymerization in the presence of H_2_SO_4_ and HCl, studies of cyclic voltammetry, Raman scattering, IR spectroscopy and photoluminescence (PL) will be shown in this paper.

Composites based on PANI and carbon nanotubes (CNTs) have been studied extensively, due to their numerous applications in various fields including pharmaceuticals (determination of paracetamol)^10^, supercapacitors^11,12^, electrodes for dye-sensitized solar cells^13^, fuel cells^14^, actuators^15^, rechargeable batteries^16^, gas sensor^17^, photocatalysts^18^ and so one. Such applications were also reported in the case of other polyaniline-based composites, some examples being (i) polyaniline-silver nanocomposite as active material in detection of H_2_S^19^ and (ii) PANI- modified TiO_2_ as photocatalytic material for the degradation of organic pollutants^20^. To achieve of all these applications, a good understanding of the optical and electrochemical properties was necessary. Often, the synthesis methods used for the preparation of the PANI/CNTs composites were: (i) chemical polymerization of ANI in the presence of CNTs and different oxidative mixtures such as FeCl_3_^11^, K_2_Cr_2_O_7_ and H_2_SO_4_^21^, H_2_O_2_^22^ and (NH_4_)_2_S_2_O_8_^22^; (ii) ANI electropolymerization in different acid media such as HCl and H_2_SO_4_^6^ and (iii) the chemical interaction of PANI with CNTs^22^. A sustained effort was focused on understanding the optical properties of composite materials based on PANI and multi-walled carbon nanotubes as well as single-walled carbon nanotubes (SWNTs)^6,10–16,21,22^. Depending on the weight of the two entities in the repeating units of PANI, i.e., reduced (labeled R, this having benzene rings and amine groups) and oxidized (labeled O, consisting of quinoid rings and imine groups), three molecular structures were reported: leucoemeraldine (R = 1, O = 0), emeraldine (R = O = 1) and pernigraniline (R = 0, O = 1). These molecular structures for PANI in un-doped/doped states were known as leucoemeraldine base/salt (LB/LS), emeraldine base/salt (EB/ES) and pernigraniline base/salt (PB/PS). We note that the majority of studies reported to date have been performed only on SWNT samples which consisted of a mixture of 33% metallic tubes and 66% semiconducting tubes (labeled M + S-SWNTs)^6,21,22^. To a better understanding of the motivation for the topic addressed in this paper, a brief overview of the vibrational properties of PANI/M + S-SWNT composites, reported up to the present, is described in the following: (i) according to the studies of surface enhanced Raman scattering and IR absorption spectroscopy, the ANI *in-situ* chemical polymerization in the presence of CNTs and H_2_SO_4_ involves a charge transfer between the two constituents, when the generation of a PANI doped with anion radicals of the SWNT’s fragments was demonstrated^21^; (ii) significant steric hindrance effects were invoked by IR absorption spectroscopy as a result of covalent functionalization of M + S-SWNTs with PANI-salt in the presence of an HCl medium^6^; (iii) according to early IR absorption studies, the chemical interaction of the PANI-salt/M + S-SWNT composites with the NH_4_OH solution induces an internal redox reaction when a change occurs in the macromolecular compound from a semi-oxidized state to a reduced state, and the PANI-base is obtained^6^. An interesting optical property of PANI, which has been studied less frequently, is photoluminescence (PL)^23,24^. The optical methods able to detect the formation of ANI oligomers have been reported to be IR absorption spectroscopy^25,26^ and PL^27^. At present, to the best of the authors’ knowledge, there is no article that is focused on the influence of SWNTs on the PL of PANI electrosynthesized in the presence of HCl or H_2_SO_4_. We will investigate this optical property of composites based on PANI and SWNTs enriched in metallic tubes (98%, called M-SWNTs) and semiconducting tubes (99%, called S-SWNTs), respectively, in order to answer the following questions: (i) Does the ANI electropolymerization, in the presence of HCl or H_2_SO_4_ solutions, lead to the formation of longer and shorter macromolecular chains (MCs) like those of the PANI and aniline oligomers? (ii) Do SWNTs influence the weight of the longer and shorter MCs in the reaction product mass resulted by the ANI electropolymerization? and (iii) Do M-SWNTs or S-SWNTs have an identical role in the PANI PL quenching process?

## Results

### Electrosynthesis of the PANI/SWNTs composites

**Figure 1:**
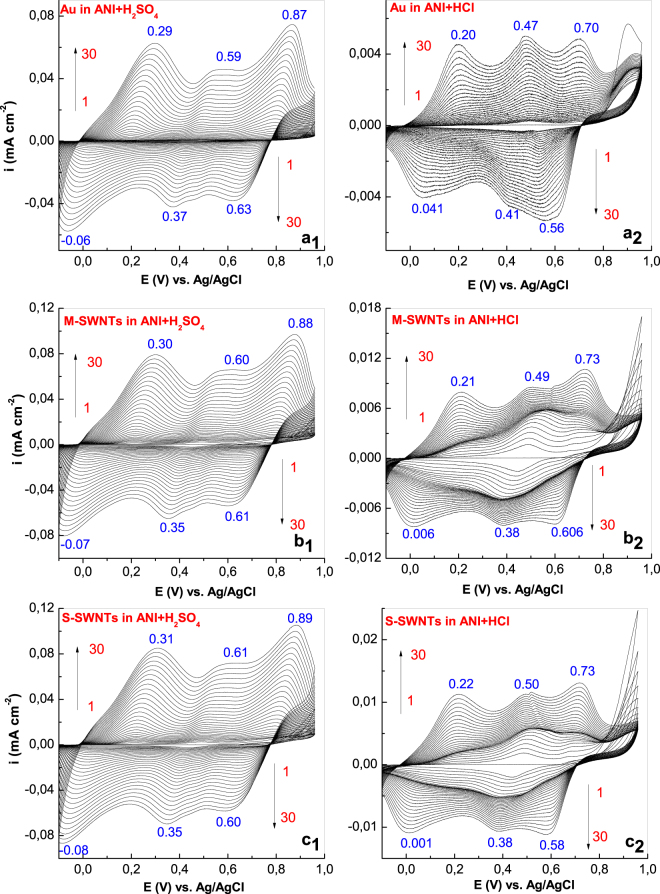
CVs describing the ANI electropolymerization, in the presence of the H_2_SO_4_ and HCl solutions, are recorded onto the blank Au electrode (**a**_**1**_,**a**_**2**_) and the Au plates covered with M-SWNT (**b**_**1**_,**b**_**2**_), and S-SWNT (**c**_**1**_,**c**_**2**_) films, respectively.

Figure 1 shows the cyclic voltammograms (CVs) of the ANI electropolymerization in the two aqueous solutions of HCl and H_2_SO_4_ recorded onto the blank Au electrode and Au plates covered with M-SWNT and S-SWNT films, respectively. According to Fig. 1, in all cases, as increasing of CVs number, a growth in the anodic and cathodic current densities was observed. In the case of the H_2_SO_4_ solution, the CVs recorded onto the blank Au electrode are characterized by three oxidation maxima situated at +0.29, +0.59 and +0.87 V, and three reduction maxima peaked at −0.06, +0.37 and +0.63 V (Fig. 1a_1_). The presence of M-SWNTs and S-SWNTs induces a gradual shift of the three oxidation peaks at (i) +0.30, +0.60, +0.88 V (Fig. 1b_1_) and (ii) +0.31, +0.61 and +0.89 V (Fig. 1c_1_), respectively. This change is accompanied by a gradual shift in the reduction peaks at (i) −0.07, +0.35, +0.61 V (Fig. 1b_1_) and (ii) −0.08, +0.36 and +0.80 V, (Fig. 1c_1_), respectively. A similar behavior is reported for the samples synthesized in the HCl medium. The CVs shown in Fig. 1a_2_,b_2_ and c_2_ illustrate in the case of (i) the blank Au electrode, three oxidation maxima situated at +0.20, +0.47 and +0.70 V, and three reduction peaks at +0.041, +0.41 and +0.56 V (Fig. 1a_2_); (ii) the Au electrode covered with a M-SWNT film, the oxidation peaks localized at +0.21, +0.49 and +0.73 V, they being accompanied by three reduction peaks at +0.006, +0.36 and +0.606 V (Fig. 1b_2_), respectively; and for (iii) the Au electrode covered with a S-SWNT film, the oxidation maxima peaked at +0.22, +0.50 and +0.73 V while the three reduction maxima peaked at +0.001, +0.38, +0.58 V (Fig. 1c_2_). Supplementary Fig. 1 reveals an increase in the anodic and cathodic current densities, when the Au electrodes were covered with M-SWNT and S-SWNT films. All of these differences, observed in Fig. 1, clearly indicate the formation of PANI-salt onto the surface of the three electrodes with some changes in the reaction mechanism of the ANI electropolymerization as a result of the CNTs’ presence. A linear behavior of the semi-logarithmic dependence of the current density in the case of the second oxidation maximum with scan rate is illustrated for the six samples studied in this paper (Fig. S1 in Supplementary Information), the fact which indicates that the electron transfer is controlled by diffusion. A schematic illustration of the ANI electropolymerization in the presence of the H_2_SO_4_ and HCl solutions onto the Au electrode covered with S-SWNTs and M-SWNTs films as well as the chemical reactions mechanism involved in the preparation of the PANI/SWNTs composite films are shown in Figs S2 and S3 in Supplementary Information. A short comment concerning the chemical reactions mechanism which take place during the PANI/SWNTs composites synthesis was also included in Supplementary Information.

### Vibrational properties of the PANI/SWNTs composites

The arguments concerning the formation of PANI-salt onto the three electrodes are shown by the Raman and IR spectroscopy (Figs S4–S11 in Supplementary Information). These demonstrate that the ANI electropolymerization in the presence of S-SWNTs and M-SWNTs leads to (i) S-SWNTs and M-SWNTs covalently functionalized with PANI-ES and PANI-LS, respectively, and (ii) ANI TR and TT. The Raman spectrum of S-SWNTs (Fig. S4a_1_ and b_1_ in Supplementary Information) is characterized in the spectral range 100–1700 cm^−1^, by three bands situated at 171, 1274 and 1570–1593 cm^−1^, which were assigned to the radial breathing mode (RBM), disorder state or the defects induced on CNTs (D band) and the tangential mode (TM)^28^. According to Fig. S4a_1_–a_4_ and b_1_–b_4_ (Supplementary Information), as increasing of CVs number recorded onto the Au electrode covered with a S-SWNT film during the ANI electropolymerization, a decrease in the relative intensity of the Raman line assigned to the RBM is observed, simultaneously with a gradual increase in the relative intensity of the PANI-ES Raman lines; a similar behavior was also reported in the case of PANI electrosynthetized onto the Au electrode covered with a M + S-SWNT film^6^. As shown in previous studies of Raman spectroscopy reported on PANI in a doped state, the main Raman lines of PANI-ES are situated at 418, 520, 1170, 1243, 1327–1367, 1500, 1573 and 1627 cm^−1^, they being assigned to the vibrational modes of out-of-plane ring deformation, out-of-plane C-H wagging, C-H bending in the benzene (B) ring, C-N stretching + B ring deformation, protonated structure, C=N stretching + C-H bending in the B ring, C=C stretching in the Q ring + C-C stretching in the B ring and C-C stretching in the B ring + C-H bending in the B ring, respectively^29,30^. At present, it is well known that the chemical interaction of PANI-ES with a NH_4_OH solution leads to the formation of PANI-EB, a polymer that is characterized by Raman lines situated at 410, 515, 1160, 1217, 1303, 1372, 1480 and 1583 cm^−1^. These Raman lines are assigned to the vibrational modes of out-of-plane B ring deformation, in-plane Q ring deformation, C-H bending in the Q ring, C-N stretching + B ring deformation + C-H bending in the B ring, C-H bending in the Q ring, C-C stretching in the Q ring + C-H bending in the B ring, C=N stretching + C-H bending in the B ring and C-C stretching in the B ring + C=C stretching in the Q ring, respectively^31^. Several differences in the Raman lines positions of PANI-salt synthesized onto Au electrodes covered with a S-SWNT film, compared with PANI-salt synthesized in the absence of S-SWNTs^6^, can be observed in Fig. S4a (Supplementary Information) as follows: (i) a down-shift of the Raman line situated in the spectral range of 1000–1200 cm^−1^ from 1170 cm^−1^^29,30^, to 1166 cm^−1^ (Fig. S4a in Supplementary Information); (ii) a modification in the position of the Raman line assigned to the C-N stretching vibrational mode from 1243 cm^−1^^29,30^, to 1260 cm^−1^ (Fig. S4 in Supplementary Information); (iii) the ratio between relative intensities of the Raman lines situated at 1593–1596 and 1570 cm^−1^ varies from 44.5 to 1 (Fig. S4a in Supplementary Information). The modification of this ratio indicates a covalent functionalization of the S-SWNT wall with PANI-salt. The successive interaction of PANI-salt covalently functionalized S-SWNTs with NH_4_OH leads to: (i) a significant decrease in the relative intensity of the PANI Raman lines situated at 410, 515, 1174, 1315–1377 and 1502 cm^−1^; (ii) the Raman lines of S-SWNTs assigned to the RBM and TM vibrational modes are situated at 179 and 1596 cm^−1^; and (iii) the higher half-width of the Raman band assigned to the S-SWNT TM. This last fact clearly indicates that successive interactions of PANI-salt covalently functionalized S-SWNTs with NH_4_OH leads to S-SWNTs covalently functionalized with a PANI-base.

The main changes induced of the ANI electropolymerization in the presence of HCl to S-SWNTs are highlighted in Fig. S4b (Supplementary Information) by: (i) a down-shift of the RBM mode from 171 to 167 cm^−1^ which is simultaneous with the increase in the relative intensity of Raman lines situated at 1570 cm^−1^; and (ii) the appearance of all Raman lines of the PANI-salt. In this last case, the chemical interaction of PANI-salt covalently functionalized SWNTs with NH_4_OH leads to a down-shift of the RBM Raman line at 169 cm^−1^, while the PANI Raman lines are peaked at 1174, 1330–1371, and 1502 cm^−1^.

Figures S5 and S6 in Supplementary Information show the modifications induced to the M-SWNTs’ Raman spectra of the ANI electropolymerization in the presence of the H_2_SO_4_ and HCl aqueous solutions. In comparison with S-SWNTs, the Raman spectrum of M-SWNTs (Figs S5, S6 in Supplementary Information) shows a band situated at 170 cm^−1^ assigned to the RBM, a D band situated at 1304 cm^−1^ and a complex TM band with a maximum at 1571 cm^−1^, which has an asymmetrical profile to smaller frequencies as a consequence of the presence of the Raman line situated at 1535 cm^−1^, known as a Breit–Wigner–Fano (BWF) component, which was assigned to the electron-phonon interaction^28^.

The ANI electropolymerization in the presence of the H_2_SO_4_ and HCl aqueous solutions (Figs S5, S6 in Supplementary Information) induces in the M-SWNTs’ Raman spectrum the following variations: (i) an up-shift of the RBM Raman line from 170 cm^−1^ (Figs S5a, S6a in Supplementary Information) to 172 cm^−1^ (Fig. S5b in Supplementary Information) and 183 cm^−1^ (Fig. S6b,c in Supplementary Information) simultaneously with a gradual decrease in the relative intensity until its disappearance, when the 30 CVs were recorded onto the working electrode (Figs S5c, S6d in Supplementary Information); (ii) a change in the profile of the TM Raman line, as a result of the decrease in the relative intensity of the BWF component simultaneously with a shift in the Raman line from 1571 cm^−1^ to 1601–1602 cm^−1^ (Figs S5c, S6d in Supplementary Information); and (iii) the increase in the relative intensity of PANI-salt Raman lines situated at 1174–1176, 1336–1383, 1507–1515 and 1601 cm^−1^. These changes indicate breaking of the M-SWNTs bundles into individual tubes subsequent to a covalent functionalization with PANI-salt (called in the following, M-SWNTs/PANI^+^HSO_4_^−^ and M-SWNTs/PANI^+^Cl^−^). The interaction of PANI-salt covalently functionalized M-SWNTs with the 1 M NH_4_OH solution leads to: (i) an additional shift of the RBM Raman band, at 176 cm^−1^ (Fig. S6d in Supplementary Information) and 179 cm^−1^ (Fig. S6e in Supplementary Information); (ii) a partial recovery of the TM Raman band profile; (iii) the D band maximum is localized at 1313 cm^−1^ (Fig. S6e in Supplementary Information); and (iv) a significant decrease in the relative intensity of the PANI Raman lines until their disappearance. As shown in Fig. S6d and e (Supplementary Information), the Raman spectra shows only one Raman line of small intensity peaked at 1161–1162 cm^−1^, assigned to the vibrational mode of C-H bending in the quinoid ring of the PANI-base^30^. The Raman line at 1161–1162 cm^−1^, indicates unequivocally the presence of a PANI-base onto the M-SWNTs’ surface, after the interaction of the PANI-salt covalently functionalized M-SWNTs with the 1 M NH_4_OH solution. A plausible hypothesis which may explain the small intensity of the Raman line situated at cca. 1161–1162 cm^−1^ as well as the absence of other Raman lines of the PANI-base is the formation of PANI-LB, which is difficult to reveal in Raman spectra recorded at an excitation wavelength of 676 nm^30^. Additional information, which sustains the formation of PANI-LB, is shown by IR spectroscopy in Supplementary Information section (see, Figs S7–S10 and related comments).

### Photoluminescence properties of the PANI/SWNTs composites

**Figure 2:**
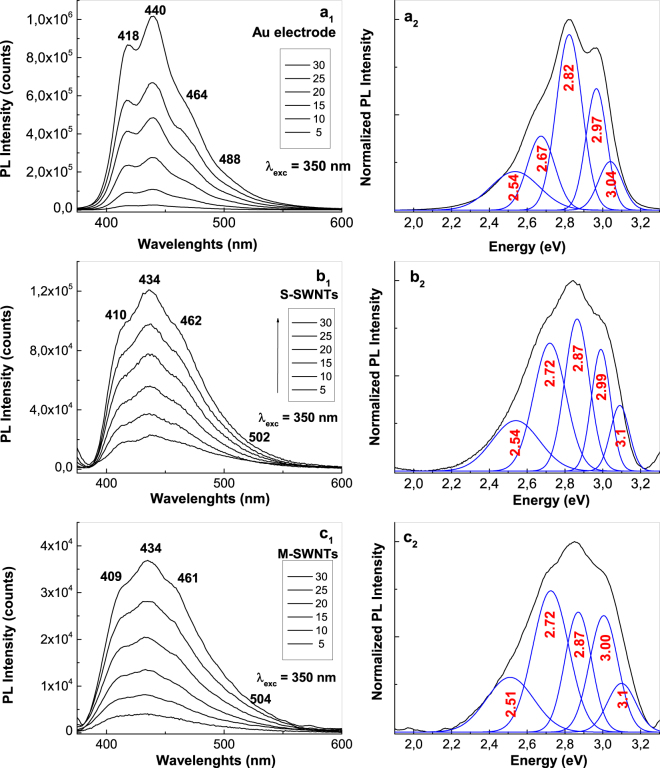
The PL spectra of PANI doped with HSO_4_^−^ ions electro-synthesized onto the blank Au electrode (**a**_**1**_) and the Au plates covered with a film of S-SWNTs (**b**_**1**_) and M-SWNTs (**c**_1_) during 5, 10, 15, 20, 25 and 30 CVs. De-convolution of the PL spectra recorded after 30 CVs onto the blank Au electrode (**a**_**2**_) and the Au plates covered with a film of S-SWNTs (**b**_**2**_) and M-SWNTs (**c**_**2**_).

**Figure 3:**
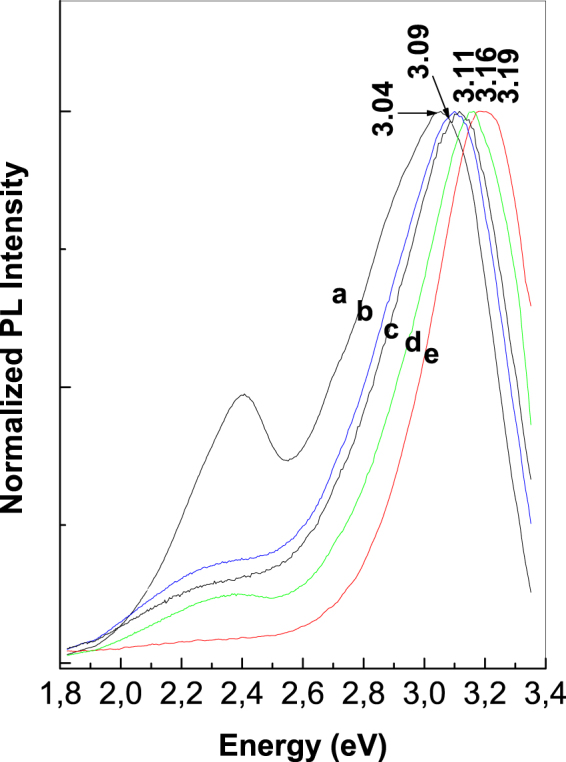
The PL spectra of the powder (**a**) and ANI TR films with thicknesses of 256 (**b**), 192 (**c**), 92 (**d**) and 32 nm (**e**).

As observed in Fig. 2a_1_, as the CVs’ number increases, a gradual increase in the PL intensity of PANI doped with HSO_4_^−^ ions takes place. After 30 CVs are recorded onto the blank Au electrode, the PL spectra of PANI doped with HSO_4_^−^ ions, show five emission bands peaking at 408 nm (3.04 eV), 418 nm (2.97 eV), 440 nm (2.82 eV), 464 nm (2.67 eV) and 488 nm (2.54 eV). The last four PL bands were assigned to the electronic transitions of small MCs of tetramer (TT) type^27^, the reduced entities of the PANI repeating units^6,27^, the oxidative entities of the repeating units of PANI in an un-doped state^23^ and the oxidative entities that contain semi-quinoid structures in the repeating units of PANI doped with HSO_4_^−^ ions^6^ (Fig. 2a_1_,a_2_). The co-existence of PL bands at 2.67 and 2.54 eV indicates the presence of a PANI that is partially doped. With respect to the PL band with a maximum at 3.04 eV, this was assigned by us to the ANI trimmers (TR). This assignment is achieved taking into account the PL spectrum of ANI TR (known as N,N′-diphenyl-1,4-phenylenediamine), in powder and the thin film state. The ANI TR films were deposited onto gold supports from a solution of ANI TR in CH_3_CN with a concentration of 1 mg/ml, when layers with different thicknesses of 32, 92, 192 and 256 nm, were obtained. According to Fig. 3, the PL spectra of the powder and films of ANI TR, with thicknesses of 256, 192, 92 and 32 nm, highlight an intense emission band with the maximum varying from 3.04 eV (Fig. 3, curve a) to 3.09 eV (Fig. 3, curve b), 3.11 eV (Fig. 3, curve c), 3.16 eV (Fig. 3, curve d) and 3.19 eV (Fig. 3, curve e).

Returning to Fig. 2, regardless of the number of CVs recorded onto the working electrode, a decrease in the PL intensities of the PANI electrosynthesized onto Au plates covered with films of S-SWNTs and M-SWNTs was observed in Fig. 2b_1_,c_1_. After 30 CVs, a decrease from 10^6^ to 1.2 × 10^5^ and 3.8 × 10^4^ counts was reported when PANI was deposited onto the blank Au electrode and Au plates covered with S-SWNT and M-SWNT films, respectively (Fig. 2a_1_,b_1_,c_1_). A careful analysis of Fig. 2a_2_,b_2_ and c_2_ indicates: (i) an up-shift in the ANI TT and TR PL band, from 2.97 and 3.04 eV to 3.00 and 3.1 eV, respectively; (ii) an up-shift of the PL band assigned to the PANI reduced and oxidized entities, from 2.82 and 2.67 eV to 2.87 and 2.72 eV, respectively; and (iii) a down-shift of the PL band assigned to the oxidative entities of PANI in doped state, from 2.54 to 2.51 eV, when the polymer was synthesized onto the blank Au electrode and Au plate covered with the M-SWNT film, respectively. In addition, an increase in the relative intensities of the PL bands assigned to the oxidative entities of PANI in un-doped and doped states, respectively, was also remarked in Fig. 2a_2_,b_2_ and c_2_.

**Figure 4:**
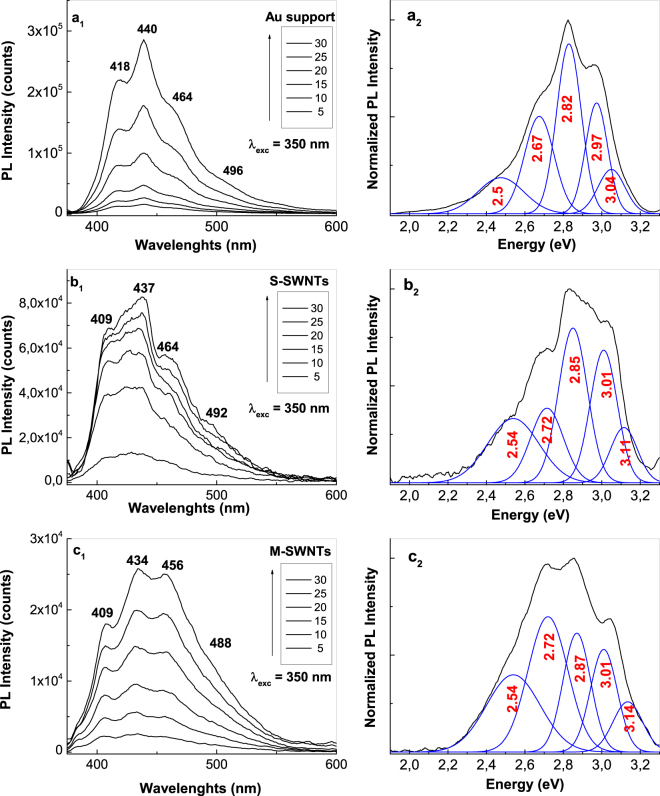
The PL spectra of PANI doped with Cl^−^ ions electrosynthesized onto the blank Au electrode (**a**_**1**_) and the Au plates covered with a film of S-SWNTs (**b**_**1**_) and M-SWNTs (**c**_**1**_) during 5, 10, 15, 20, 25 and 30 CVs. De-convolution of the PL spectra recorded after 30 CVs onto the blank Au electrode (**a**_**2**_) and the Au plates covered with a film of S-SWNTs (**b**_**2**_) and M-SWNTs (**c**_**2**_).

**Figure 5:**
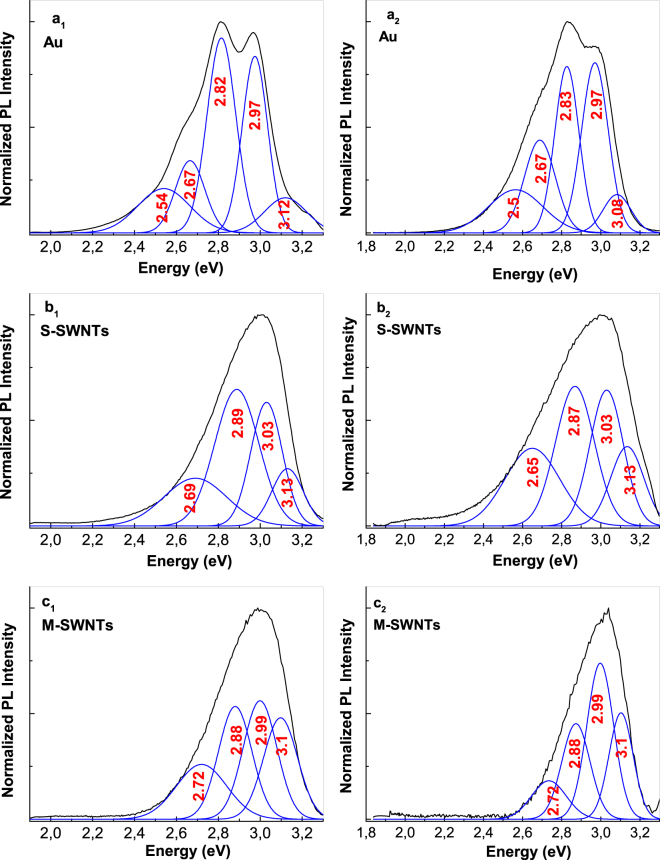
The de-convolution of normalized PL spectra of the samples of PANI doped with HSO_4_^−^ and Cl^−^ ions, respectively, electrosynthesized during the 30 CVs onto the blank Au electrode (**a**_**1**_ and **a**_**2**_) and the Au plates covered with films of S-SWNTs (**b**_**1**_ and **b**_**2**_) and M- SWNTs (**c**_**1**_ and **c**_**2**_), after the interaction with the 1 M NH_4_OH solution.

**Figure 6:**
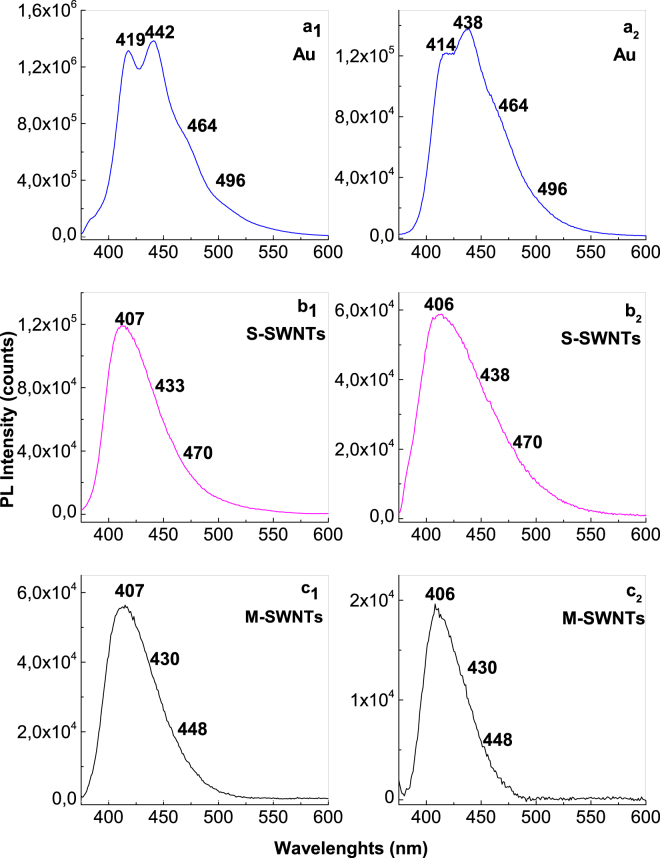
The PL spectra of the samples of PANI doped with HSO_4_^−^ and Cl^−^ ions, respectively, electrosynthesized during the 30 CVs onto the blank Au electrode (**a**_**1**_ and **a**_**2**_) and the Au plates covered with films of S-SWNTs (**b**_**1**_ and **b**_**2**_) and M-SWNTs (**c**_**1**_ and **c**_**2**_), after the interaction with the 1 M NH_4_OH solution.

**Figure 7:**
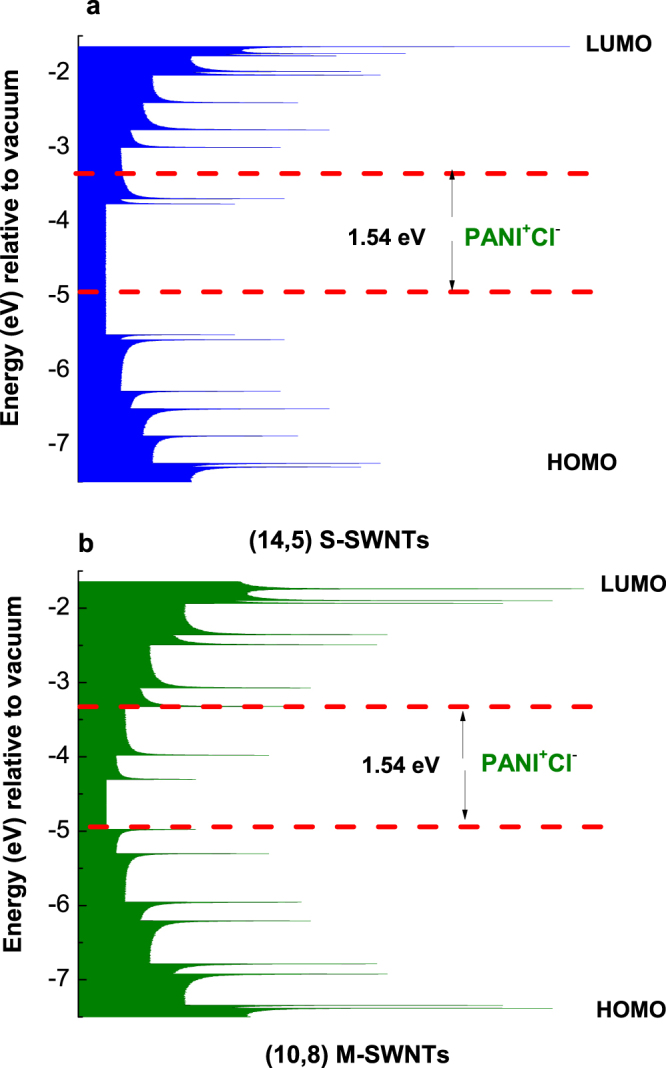
Diagram of electronic energy levels of S-SWNTs (**a**), M-SWNTs (**b**) and PANI doped with Cl^−^ ions (red lines).

A similar behavior to that shown in Fig. 2 is highlighted in the case of PANI electrosynthesized in the presence of HCl (Fig. 4) as follows: (i) as growing of CVs numbers recorded onto the blank Au support, a progressive increase in the PANI PL intensity until 2.97 × 10^5^ counts is reported in the case of PANI doped with Cl^−^ ions; (ii) in the presence of CNTs, a decrease in the PL intensity of PANI doped with Cl^−^ ions occurs from 2.97 × 10^5^ counts to 8.2 × 10^4^ and 2.57 × 10^4^ counts, when the PANI is electrosynthetized onto the blank Au electrode and Au plates covered with S-SWNT and M-SWNT films, respectively; in the presence of M-SWNTs and S-SWNTs, an up-shift in the PL bands belonging the electronic transitions of ANI TT and TR, from 2.97 and 3.04 eV (Fig. 4a_2_) to 3.01 eV (Fig. 4b_2_,c_2_) and 3.11–3.14 eV (Fig. 4b_2_,c_2_), respectively, is reported; and (iv) an up-shift in all the electronic transitions of PANI from 2.5, 2.67 and 2.82 eV (Fig. 4a_2_) to 2.54, 2.72 and 2.85 eV (Fig. 4b_2_) and 2.54, 2.72 and 2.87 eV (Fig. 4c_2_) is induced by the S-SWNTs and M-SWNTs. Considering the results shown in Figs 2 and 4, a PANI-salt PL quenching role is invoked that plays out on both the M-SWNTs and S-SWNTs. Figure 5 shows the PL spectra of the samples obtained onto the three working electrodes in the presence of H_2_SO_4_ and HCl after the interaction with the NH_4_OH solution. The interaction of NH_4_OH with the reaction products resulted from the ANI electropolymerization, in the presence of the H_2_SO_4_ or HCl solutions, when these were deposited onto the Au plates covered with S-SWNT and M-SWNT films, induced a significant change in the PL spectra profile (Fig. 5). Comparing Figs 2, 4 and 5, the interaction of the reaction products with the NH_4_OH solution induces, in the case of: (i) the blank Au electrode, a shift in PL bands belonging the ANI TR electronic transition from 3.04 eV (Figs 2a_2_, 4a_2_) to 3.08 eV (Fig. 5a_2_) and 3.12 eV (Fig. 5a_1_), respectively; (ii) the samples electrosynthesized in the presence of H_2_SO_4_ or HCl which were deposited onto the Au plates covered with the S-SWNT and M-SWNT films, a disappearance of the PL band assigned to the oxidized entities of PANI in doped state (Fig. 5b_1_,b_2_,c_1_,c_2_). For the same CVs number recorded onto the blank Au support and Au plates covered with S-SWNT and M-SWNT films, a different decrease in the intensity of PL spectra is noted in the last two cases (Figs 2 and 4). As observed in Fig. 6, a similar behavior is observed in the case of the samples after their interaction with the 1 M NH_4_OH solution. This fact highlights a PANI PL quenching effect more intense in the case of M-SWNTs in comparison with S-SWNTs. This behavior can be explained taking into account the diagrams of the electronic energy levels of S-SWNTs, M-SWNTs and PANI (Fig. 7), calculated according to our previously papers^32–35^. Briefly, according to our Raman spectroscopy studies, Figs S4b and S6b,c show the Raman spectra of S-SWNTs and M-SWNTs before and after electrochemical polymerization of aniline in the presence of the HCl aqueous solution, when in the low frequencies range a Raman line, assigned to the radial breathing mode (RBM) vibrational mode, with the maximum at 167 and 183 cm^−1^, respectively, was observed. Taking into account the relationship between the RBM Raman line and carbon nanotubes diameter ω_RBM_ (cm^−1^) = 224/d (nm)^36^, we have calculated that in the case of the PANI/S-SWNTs and PANI/M-SWNTs samples, the carbon nanotubes diameters were equal with 1.42 nm and 1.34 nm, respectively. The chirality of the S-SWNTs and M-SWNTs was assessed using the Kataura plot, this being (14,5) and (10,8), respectively. Using the work function equal with −4.66 eV^37^, the energy levels of (14,5) S-SWNTs and (10,8) M-SWNTs were calculated and represented in Fig. 7. Thus, Fig. 7 shows the diagrams of the electronic energy levels of S-SWNTs and M-SWNTs as well as PANI doped with Cl^−^ ions. The optical band gap of PANI doped with Cl^−^ ions and the onset potential for the oxidation of macromolecular compound were equal with 1.54 eV^35^ and 0.47 eV, respectively. The PANI PL quenching mechanism takes into account that the excitons resulted onto the MCs of PANI doped with Cl^−^ ions, under optical excitation, are dissociated into electrons and holes. Further, the electrons are collected of LUMO levels of S-SWNTs with the chirality (14,5) and M-SWNTs with the chirality (10,8) and successively passed on the levels with smaller energy values until to the lower LUMO level. According to Fig. 7, the superposition of the HOMO levels of the two constituents, i.e. macromolecular compound and CNTs, is observed only in the case of PANI doped with Cl^−^ ions and M-SWNTs. This fact allows the collecting of the electrons both in the valence band of PANI doped with Cl^−^ ions and M-SWNTs. The difference of energy between HOMO levels of PANI doped with Cl^−^ ions and S-SWNTs will induced a recombination of electrons with the holes from valence band of PANI doped with Cl^−^ ions. This fact will induce a PANI PL quenching with higher efficiency in the case of M-SWNTs in comparison with S-SWNTs. In addition to this mechanism, the PANI PL quenching is also due to the increase in the exciting light absorbance, as a consequence of the presence of the S-SWNTs and M-SWNTs.

**Figure 8:**
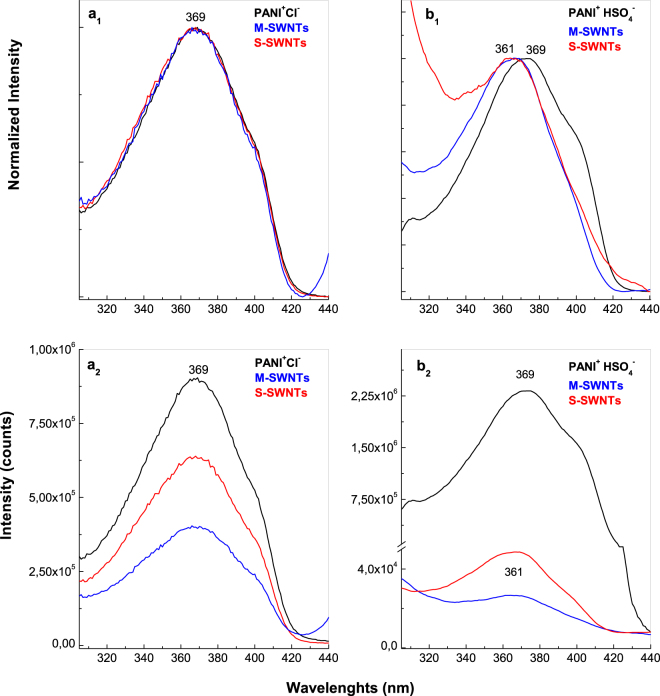
The PL excitation spectra of: PANI doped with Cl^−^ ions (black curves in **a**_**1**,_**a**_**2**_) and PANI doped with HSO_4_^−^ ions (black curves in **b**_**1**_,**b**_**2**_), the S-SWNTs/PANI doped with HSO_4_^−^ ions (red curves in **b**_**1**_,**b**_**2**_) and S-SWNTs/PANI doped with Cl^−^ composites (red curves in **a**_**1**_,**a**_**2**_) and (iii) the M-SWNTs/PANI doped with HSO_4_^−^ ions (blue curves in **b**_**1**_,**b**_**2**_) and M-SWNTs/ PANI doped with Cl^−^ ions composites (blue curves in **a**_**1**_,**a**_**2**_). All spectra were recorded at the emission wavelength of 460 nm.

The photoluminescence excitation (PLE) spectra of the six samples are shown in Fig. 8. As common features of these spectra, we note that: (i) both in the case of PANI doped with HSO_4_^−^ ions and PANI doped with Cl^−^ ions, the PLE spectra are characterized by a band with maximum at 369 nm; and (ii) a decrease in the relative intensity of the PLE spectra of PANI doped with HSO_4_^−^ ions and PANI doped with Cl^−^ ions, respectively, when the electrochemical polymerization of aniline takes place onto Au electrodes covered with S-SWNTs and M-SWNTs films is observed in Fig. 8. The significant differences in PLE spectra both in the PLE band maximum position and profile are observed in the case of the PANI doped with HSO_4_^−^ ions synthetized onto the blank Au electrode and the Au plates covered with the M-SWNTs and S-SWNTs films. An up-shift of PLE band of PANI doped with HSO_4_^−^ ions from 369 nm (3.36 eV) to 361 nm (3.43 eV) is observed in the presence of M-SWNTs and S-SWNTs. In our opinion, this variation is a consequence of a change of the ratio between electronic transitions of the reduced and semi-oxidized entities of PANI when the ANI electropolymerization takes place in the presence of H_2_SO_4_ solution onto Au electrode and Au plates covered with the M-SWNTs and S-SWNTs films. This sentence takes into account the values of the ratios between the relative intensities of the PANI reduced and semi-oxidized entities PL bands (I_PL-R_/I_PL-SQ_). According to Figs 2a_2_,b_2_,c_2_ and 4a_2_,b_2_,c_2_, the I_PL-R_/I_PL-SQ_ ratio has the value in the case: (i) PANI doped with HSO_4_^−^ ions and its composites with S-SWNTs and M-SWNTs equal with 4.6, 3 and 2.2 and (ii) PANI doped with Cl^−^ ions and its composites with S-SWNTs and M-SWNTs equal with 4.6, 2.4 and 1.5. These changes must be understood by the different number of benzene and quinoid rings in macromolecular structures of composite materials shown in Fig. S3 as well as their weight in the electrochemical reactions products mass.

## Discussion

Using photoluminescence (PL) and IR spectroscopy, this work highlights for the first time that the electrochemical polymerization of aniline in the presence of H_2_SO_4_ and HCl solutions leads both to PANI-salt and to short macromolecular chains of the type aniline trimers (TR) and tetramers (TT). Regardless of the acid medium used for the ANI electropolymerization onto the blank Au electrode, the electrochemical polymerization reaction product shows five emission bands at 407, 418, 440, 464 and 496 nm which were assigned to the electronic transitions of small MCs of the ANI TR and TT types, the reduced entities of the PANI repeating units and the oxidative entities belonging to the repeating units of PANI in the un-doped and doped states, respectively. Additional optical evidence for ANI TT and TR consists of the IR absorption bands peaking in the spectral range 700–742 cm^−1^ which are assigned to the vibrational mode of C-N=C bending. In comparison with other macromolecular compounds as poly(para-phenylenevinylene) for which formation of short MCs is an experimental fact well known^33^, the formation of ANI TR and TT as secondary products of the synthesis of PANI-salt is a new result which has not been reported so far.

A reaction mechanism is reported for the ANI electrochemical polymerization in the presence of H_2_SO_4_ and HCl solutions using Au plates covered with M-SWNTs or S-SWNTs as working electrodes. The polymerization reaction products were confirmed by IR spectroscopy and PL. In this context, the correlated studies of PL and FTIR spectroscopy have demonstrated that the ANI electropolymerization in the presence of the S-SWNTs and M-SWNTs leads to a decrease in ANI TT weight in the reaction product mass consisting in S-SWNTs and M-SWNTs covalently functionalized with PANI-ES and PANI-LS, respectively. The linear behavior of the semi-logarithmic dependence of the current density for the second oxidation maximum of aniline with scan rate indicates that the electron transfer is controlled by a diffusion process.

A new challenge in the understanding of optical properties of the PANI doped with Cl^−^ and HSO_4_^−^ ions and its composites with M-SWNTs and S-SWNTs is the PLE studies. In this context, the up-shift of the polymer PLE band in the presence of carbon nanotubes was explained on the base of different number of benzene and quinoid rings in macromolecular structures of composite materials resulted according to Fig. S3 as well as their weight in the electrochemical reactions products mass.

Using Raman scattering and FTIR spectroscopy, we demonstrated that the chemical interaction of PANI-ES/PANI-LS covalently functionalized S-SWNTs and M-SWNTs with the 1 M NH_4_OH solution leads to PANI-EB/PANI-LB covalently functionalized S-SWNTs and M-SWNTs, respectively.

According to S. Ghatak *et al*., the PL studies reported on the PANI/SWNTs, PANI/DWNTs and PANI/MWNTs composites were carried out using carbon nanotubes consisting a mixture of 33% metallic tubes and 66% semiconducting tubes^7^. In this work, it is highlighted in the preview the influence of the SWNTs highly separated in metallic tubes (M-SWNTs, 98%) and semiconducting tubes (S-SWNTs, 99%) on PL of the PANI doped with Cl^−^ and HSO_4_^−^ ions. The presence of S-SWNTs and M-SWNTs induces a PANI PL quenching process. The proposed mechanism to explain this process takes into account the diagram of electronic energy levels of the constituents of the PANI-salt/S-SWNTs and PANI-salt/M-SWNTs composites and their different de-excitation ways.

## Methods

### Electrosynthesis of PANI/M-SWNTs and PANI/S-SWNTs

The M-SWNTs and S-SWNTs were purchased from the NanoIntegris Company. The high-resolution transmission electron microscopy (HRTEM) images of M-SWNTs and S-SWNTs are shown in Fig. S11. ANI, H_2_SO_4_, HCl, NH_4_OH, CH_3_CN and N,N′-diphenyl-1,4-phenylenediamine were purchased from Sigma Aldrich. The ANI electrochemical polymerization was performed according to the protocol reported by Baibarac *et al*.^6^. In the present electrochemical setup, a one-compartment cell was used with three electrodes where the auxiliary electrode, the reference electrode, and the working electrode consisted of a spiral Pt wire, a Ag/AgCl electrode and a blank Au electrode or an Au plate covered with M-SWNT and S-SWNT films, respectively, with a thickness of 100 nm. The deposition of the S-SWNT and M-SWNT films onto Au plates was carried out by drop casting method. The thickness of the S-SWNT and M-SWNT films onto Au plates was of 120 nm. The assessing of the carbon nanotubes films thicknesses was carried out using our previously procedure^36^. A 0.05 M ANI and 0.5 M H_2_SO_4_ or 0.5 M HCl solution was prepared for the electrosynthesis of PANI-salt on a blank Au electrode or on Au plates covered with M-SWNT and S-SWNT films, respectively. Depending on the acid medium, i.e., H_2_SO_4_ or HCl, a PANI doped with the HSO_4_^−^ or Cl^−^ ions, respectively, resulted. The ANI electropolymerization was performed in the potential range (−100; +950) mV with a sweep rate of 100 mV/s.

According to scanning electron microscopy (SEM) images shown in Fig. S12, the electrochemical polymerization of aniline has induced at a complete deposition of PANI doped with HSO_4_^−^ and Cl^−^ ions onto the S-SWNTs and M-SWNTs surface. The interaction of the PANI-salt and the PANI-salt/M-SWNT and PANI-salt/S-SWNT composites with the 1 M NH_4_OH solution at a time of 5 min. was carried out in order to de-doping of PANI-salt.

The cyclic voltammograms (CVs) were recorded using a pontentiostat/galvanostat Voltalab 80 from Radiometer Analytical.

### Optical characterizations

The PL spectra of PANI and their composites synthesized in this paper were recorded using a Horiba Jobin Yvon Fluorolog 3-2.2.1 spectrometer in a right-angle geometry, at an excitation wavelength of 350 nm and room temperature. As is well known, for the ANI electrochemical polymerization can be used various electrodes as ITO, Au, Pt, etc. The choice of Au electrodes for the electrochemical synthesis of PANI and its composites with M-SWNTs and S-SWNTs was made from the same reasoning as in the case of Pt support, namely to avoid the background contribution of PL of the ITO substrate^37^. Any influence of the Au electrodes used for the electrochemical synthesis of PANI and its composites with M-SWNTs and S-SWNTs was observed to be induced in PL spectra reported in this work.

The Raman spectra of the composites were recorded in backscattering geometry at the excitation wavelengths of 1064 and 676 nm using a FT Raman spectrophotometer from Bruker, model RFS100S, and a Raman spectrophotometer from Horiba Jobin Yvon, model T64000, equipped with a Kr laser.

The IR absorption spectra were recorded with a FTIR spectrophotometer from Bruker, Vertex 70 model, using the attenuated total reflection (ATR) accessory with a diamond crystal.

The HRTEM images of S-SWNTs and M-SWNTs as well as their composites with PANI doped with Cl^−^ ions were recorded with a Jeol JSM300 F field emission gun transmission electron microscope (FEGTEM).

The SEM images of S-SWNTs and M-SWNTs as well as their composites with PANI doped with HSO_4_^−^ and Cl^−^ ions were recorded with Tescan Lyra III XMU scanning electron microscope.

## Electronic supplementary material

Supplementary Information
