# Supplementary material for: Polyaniline photoluminescence quenching induced by single-walled carbon nanotubes enriched in metallic and semiconducting tubes
Source: Sci Rep. 2018 Jun 22;8:9518. doi: 10.1038/s41598-018-27769-4 (PMC6015054; doi:10.1038/s41598-018-27769-4)
Supplement: Supplementary file 1 — Supplementary Information [file 41598_2018_27769_MOESM1_ESM.docx]

**Supplementary Information**

**Polyaniline Photoluminescence Quenching Induced by Single-Walled Carbon Nanotubes Enriched in Metallic and Semiconducting Tubes**

Mihaela Baibarac^1*^, Adelina Matea^1,2^, Monica Daescu^1^, Ionel Mercioniu^1^, Sophie Quillard^3^, Jean-Yves Mevellec^3^ and Serge Lefrant^3^

^1^Laboratory of Optical Processes in Nanostructured Materials, National Institute of Materials Physics, Bucharest, P.O. Box MG-7, R077125, Romania

^2^Faculty of Physics, University of Bucharest, 405A Atomistilor, Bucharest, P.O. Box MG-1, 077125 Romania

^3^Institut des Matériaux “Jean Rouxel”, 2 rue de la Houssinière, B.P. 32229, F-44322, Nantes cedex 3, France

*E-mail : barac@infim.ro


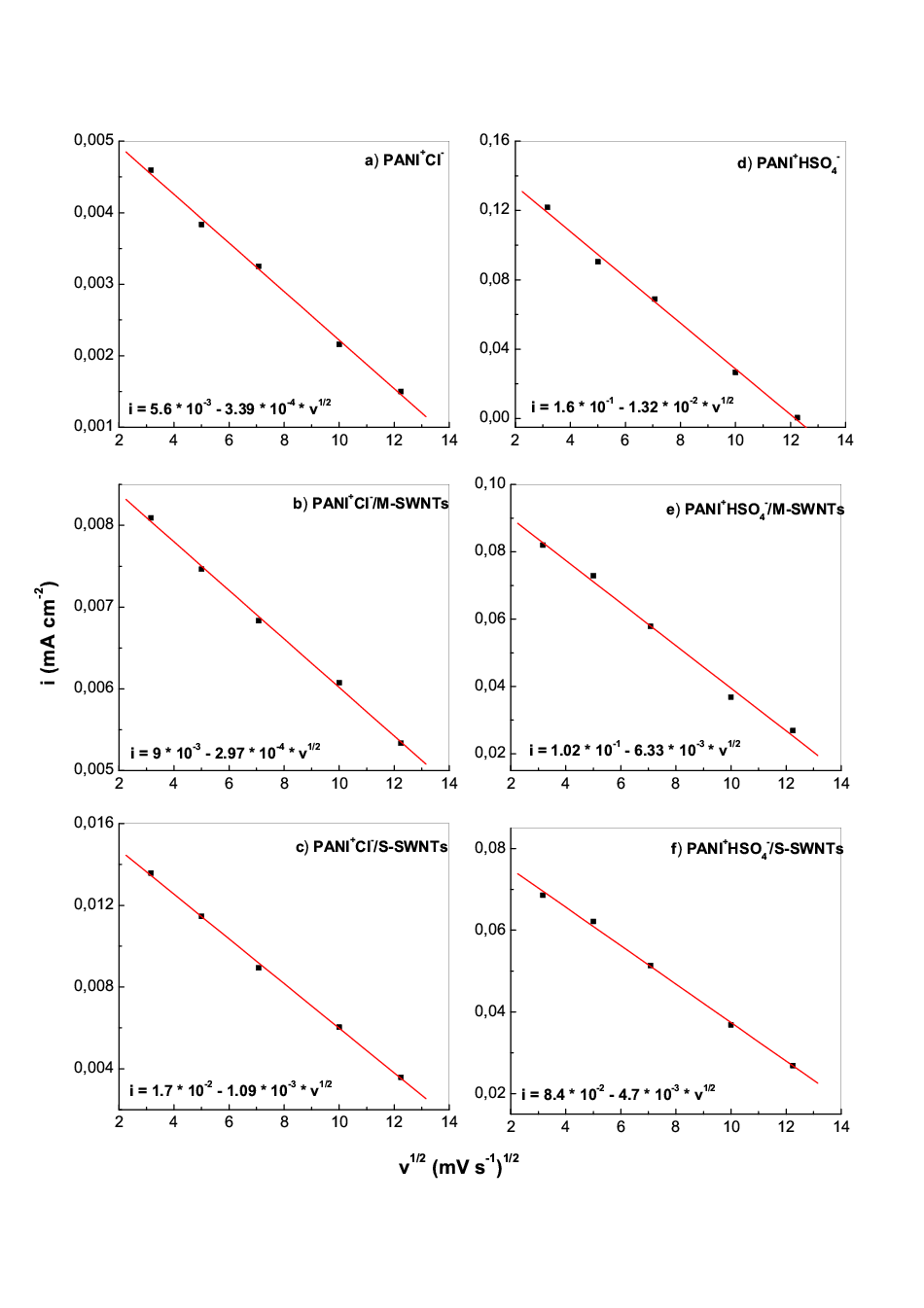


**Figure S1** The semi-logarithmic dependence of the current density of the second oxidation maximum, recorded during the anodic scanning from -100 to +950 mV vs. Ag/AgCl and after the ten cyclic voltammogram, versus the scan rate for **a**) PANI doped with $\mathrm{Cl}^{-}$ ions, **b**) composite based on M-SWNTs and PANI doped with $\mathrm{Cl}^{-}$ ions, **c**) composite based on S-SWNTs and PANI doped with $\mathrm{Cl}^{-}$, **d**) PANI doped with $\mathrm{HSO}_{4}^{-}$ ions, **e**) composite based on S-SWNTs and PANI doped with $\mathrm{HSO}_{4}^{-}$ ions, and **f**) composite based on M-SWNTs and PANI doped with $\mathrm{HSO}_{4}^{-}$ ions.

According to Figure S1, a linear behavior of the semi-logarithmic dependence of the current density in the case of the second oxidation maximum with scan rate is illustrated for the six samples studied in this paper, the fact which indicates that the electron transfer is controlled by diffusion.

**Figure S2** Schematic illustration of the aniline electrochemical polymerization process in the presence of the H_2_SO_4_ and HCl solutions onto the Au electrode covered with S-SWNTs and M-SWNTs films.

A) Reactions of the aniline cation radical with SWNTs

[Aniline covalently functionalized SWNTs]

……..


B) Reactions of the PANI macromolecular chain with SWNTs

……..

**Figure S3** Schema of the two reactions mechanisms, via the **A** and **B** ways, involved in the

PANI/SWNTs composites synthesis.

A short comment concerning the mechanism of the chemical reactions which take place during the PANI/SWNTs composites synthesis is necessary. Two chemical mechanisms are envisaged to be developed during the electrochemical polymerization of aniline onto the Au electrode covered with a SWNTs film. The first reaction mechanism (the **A** way in Figure S2) takes into account that the ANI oxidation reaction leads to the formation of a radical cation which reacts with carbon nanotubes when, a covalently functionalization of SWNTs with aniline took place, according to Figure S2. Further, the interaction of ANI covalently functionalized SWNTs with the ANI radical cation, generated in previously stage, induces the formation of the ANI dimers and the latter to large macromolecular chains of the type PANI onto the SWNTs surface, when composites of the type SWNTs covalently functionalized with PANI-LB (labeled as PANI-LB/SWNTs in Figure S2) result. The successively oxidation reaction of the PANI-LB/SWNTs composite leads to the transformation of the reduced entities of polymer into semi-oxidized ones, with the formation of the PANI-EB/SWNTs composite. As a consequence of the presence of the acid medium, a protonation reaction of PANI-EB occurs, which induces the formation of the PANI-ES/SWNTs composites according to the **A** way of Figure S2. The second reaction mechanism (the **B** way in Figure S2) takes into account that after the growing of the macromolecular chain of PANI-LB, its interaction with SWNTs leads to the formation of the PANI-ES/SWNTs composites.

|  |  |
| --- | --- |

**Figure S4** Raman spectra (λ_exc_=1064 nm) of (**a**) PANI doped with HSO_4_^-^ ions and (**b**) PANI doped with $\mathrm{Cl}^{-}$ ions, electrosynthesized onto the Au electrode covered with a film of S-SWNTs (a_1_ and b_1_, black curves) during 10 (a_2_ and b_2_, red curves), 20 (a_3_ and b_3,_ blue curves) and 30 CVs (a_4_ and b_4,_ magenta curves). The Raman spectrum highlighted by the green color (a_5_ and b_5_) corresponds to the PANI doped with $\mathrm{HSO}_{4}^{-}$ or $\mathrm{Cl}^{-}$ ions deposited onto the S-SWNTs’ surface after the recording of 30 CVs and interaction with the 1M NH_4_OH solution.

**Figure S5** Raman spectra (λ_exc_ = 676 nm) of M-SWNTs before (**a**) and after ANI electropolymerization in the presence of the H_2_SO_4_ aqueous solutions when, 10 (**b**) and 30 CVs (**c**) were recorded. The Raman spectrum of the sample obtained after the recording of 30 CVs and successively chemically interacted with the NH_4_OH aqueous solution is shown in Fig. (**d)**.

**Figure S6** Raman spectra (λ_exc_ = 676 nm) of M-SWNTs before (**a**) and after ANI electropolymerization in the presence of the HCl aqueous solution when, 10 (**b**), 20 (**c**) and 30 CVs (**d**) were recorded. The Raman spectrum of the sample obtained after the recording of 30 CVs and successively chemically interacted with the NH_4_OH solution is shown in Fig. (**e)**.

|  |  |
| --- | --- |

**Figure S7** IR spectra of: (**a**) M-SWNTs/PANI^+^$\mathrm{HSO}_{4}^{-}$ and (**b**) S-SWNTs/PANI^+^$\mathrm{HSO}_{4}^{-}$ after the recording of 10, 20 and 30 CVs.

**Figure S8** IR spectra of the M-SWNTs/PANI^+^$\mathrm{HSO}_{4}^{-}$ composite obtained after the recording of 10 (**a**), 20 (**b**) and 30 CVs (**c**) onto the working electrode and successively interacting with the NH_4_OH solution.

**Figure S9** IR spectra of the S-SWNTs/PANI^+^$\mathrm{Cl}^{-}$ composite synthesized during 10 (**a**), 20 (**b**) and 30 CVs (**c**) on the working electrode and successively interacted with the 1M NH_4_OH solution (**d**).

**Figure S10** IR spectra of the M-SWNTs/ PANI^+^$\mathrm{Cl}^{-}$ composites synthesized by the recording of 10 (**a**), 20 (**b**) and 30 CVs (**c**) onto the working electrode and successively interacted with the 1M NH_4_OH solution (**d**).

According to our expectations, the ANI electropolymerization in the H_2_SO_4_ solution onto the Au electrode covered with a M-SWNT film induces the appearance of the PANI-salt IR absorption bands, these being situated at 824, 1146–1148, 1247–1251, 1294–1307, 1498 and 1587–1594 cm^-1^ (Figures *S*7*a*_1_–*a*_3_). These PANI-salt IR bands are assigned to the vibrational modes of out-of-plane bending of the C-H bond of the B ring p-disubstituted, (B)-NH+=(Q), C-N stretching + B ring deformation + C-H bending in the B ring, N=Q=N, C_aromatic_-N stretching in N-B-N and N=Q=N stretching, respectively.^S1, S2^ Depending on the number of CVs recorded onto the working electrode, a change in the ratio between the absorbance of IR bands situated at 1498 cm^-1^ and those situated in the spectral ranges 800–1400 and 1510–1700 cm^-1^ is noted in Figures *S*7*a*_1_–*a*_3_. In the case of S-SWNTs, Figures *S*7*b*_1_–*b*_3_ highlight significant differences in the spectral range 800–1300 cm^-1^. The two new IR absorption bands peaked at 882–888 and 1034–1046 cm^-1^ (Figures *S*7*b*_1_–*b*_3_) are assigned to the HSO_4_^-^ vibrational modes.^S3^ A consequence of their presence is the up-shift of the IR bands at 1152–1160 cm^-1^ and 1315–1319 cm^-1^ which were assigned to the vibrational modes (B)-NH+=(Q) and N=Q=N, respectively.^S2^ In our opinion, the higher absorbance of the IR bands belonging HSO_4_^-^ ions can be explained only if we accept that during the ANI electropolymerization in the presence of the H_2_SO_4_ solution onto the Au electrode covered with a S-SWNT film, both a covalent functionalization of S-SWNTs with PANI in doped state (called S-SWNTs/PANI^+^ $\mathrm{HSO}_{4}^{-}$) and a doping of S-SWNTs with $\mathrm{HSO}_{4}^{-}$ ions takes place.

The transformation of PANI-salt in the PANI-base, induces in the IR spectra an up-shift of the IR absorption bands from 1144, 1498 and 1589 cm^-1^ to 1174, 1518 and 1622 cm^-1^, respectively.^S1, S3-S7^ Figures *S*7*a*_1_–*a*_3_ and S8a highlight the shift of the IR bands situated in the spectral ranges 1100–1200 cm^-1^, 1475–1550 cm^-1^ and 1560–1650 cm^-1^ from 1146–1148, 1496–1498, 1587–1594 cm^-1^ to 1161–1174, 1509–1518, 1604–1622 cm^-1^, respectively. This behavior indicates that the interaction of the M-SWNTs/PANI+$\mathrm{HSO}_{4}^{-}$ composite with the 1M NH_4_OH solution leads to a de-doping of the PANI-salt. A similar behavior is reported in the case of the S-SWNTs/PANI+$\mathrm{HSO}_{4}^{-}$ composite. An interesting experimental fact illustrated in Figure S8 consists of the presence of a new IR band peaked at 742 cm^-1^, which was also observed in Figure S7b, as well as the change in the ratio between the absorbance of the IR bands situated in the spectral ranges 700–785 and 800–860 cm^-1^ from 0.5 to 1 and 1.6 when the interaction of the M-SWNTs/PANI+$\mathrm{HSO}_{4}^{-}$ composites synthetized by the recording of 10, 20 and 30 CVs, respectively, with the 1M NH_4_OH solution, was carried out. Depending on the number of CVs recorded during the ANI electropolymerization onto the Au electrode covered with a S-SWNT film, the following changes are reported in PANI IR spectra shown in Figure S9: i) a down-shift of the IR band from 960 to 952 cm^-1^, when the S-SWNTs/PANI^+^$\mathrm{Cl}^{-}$ composite was reported to be obtained after the recording of 10, 20 and 30 CVs. This IR band is assigned to the vibrational mode of C-H bending + deformation of the quinoid ring. ^S8^ ii) An up-shift of the IR bands assigned to the vibrational modes (B)-NH+=(Q)^S2^ and N=Q=N ^S1, S2^ from 1136 and 1294 cm^-1^ (Figure S9a) to 1145 and 1307 cm^-1^, respectively (Figure S9c). iii) The presence of a new absorbance band with the maximum situated at cca. 732 cm^-1^. The successive reaction of the S-SWNTs/PANI^+^$\mathrm{Cl}^{-}$ composite with the 1M NH_4_OH solution leads to an up-shift of the IR bands from 1145, 1500 and 1564 cm^-1^ (Figure S9c) to 1161, 1504 and 1609 cm^-1^, respectively (Figure S9d). The presence of the IR absorption band at 1161 cm^-1^ in Figure S9d indicates clearly that the S-SWNTs covalently functionalized with the PANI-base were obtained. A comment is necessary with respect to the complex IR band situated in the spectral range 700–750 cm^-1^ in Figure S9d as it has two components at 714 and 732 cm^-1^ after the interaction of the S-SWNTs/PANI^+^$\mathrm{Cl}^{-}$ composite with the NH_4_OH solution. These bands are not situated far from the IR bands calculated in the case of ANI TR and TT, which were reported as situated at 713 and 736 cm^-1^, respectively, as they being assigned to the C-N=C bending vibrational mode.^S8^ In the case of the M-SWNTs/PANI^+^$\mathrm{Cl}^{-}$ composites, the IR band assigned to the C-N=C vibrational mode of ANI TT ^S8^ is situated at 742 cm^-1^ (Figure S10). Other changes highlighted in Figure S10, as increasing the CV number during the ANI electro-polymerization onto the Au plate covered with a M-SWNT film in the presence of the HCl aqueous solution are: i) a gradual shift of the IR band from 1136 to 1144 cm^-1^ as increasing the CVs’ number recorded onto the working electrode, which is accompanied by an IR band whose maximum remains unchanged at 1164 cm^-1^; ii) a progressive increase in absorbance of the IR band peaked in the spectral range 1545–1600 cm^-1^; iii) an up-shift of the IR band, assigned to the vibrational mode in out-of plane C-H bending of the p-disubstituted B ring,^S1, S2^ from 825 to 840 cm^-1^; iv) the appearance of two IR absorption bands peaked at 684–715 and 742 cm^-1^, assigned to the vibration modes of out-of-plane C-C stretching in the benzene rings of N,N’-diphenyl-1,4-phenylenediamine^S9^ and C-N=C bending, respectively, both in the ANI TT and N,N’- diphenyl-1,4-phenylenediamine (i.e., trimmer)^S1,S8^; and v) the ratio between the absorbance of the IR bands situated in the spectral ranges 700–785 and 800–860 cm^-1^ is changed from 7.6 to 1.96 and 0.3 when the CVs’ number is equal to 10 to 20 and 30, respectively. According Figure S10d, the successive interaction of M-SWNTs/PANI^+^$\mathrm{Cl}^{-}$ with the 1M NH_4_OH solution leads to the highlighting of the PANI-LB IR absorption bands, when the most intense IR band is that situated at 1494 cm^-1^. In this last case, an enhancement of the IR bands situated in the spectral domain 650–750 cm^-1^ is remarked upon as well. These facts confirm that the ANI electropolymerization in the presence of HCl and M-SWNTs leads to a reaction product consisting of short MCs of the ANI TR and TT types as well as composites based on large MCs like the PANI-salt covalently functionalized M-SWNTs.

Summarizing all these points, we conclude that the ANI electropolymerization in the presence of S-SWNTs and M-SWNTs leads to i) S-SWNTs and M-SWNTs covalently functionalized with PANI-ES and PANI-LS, respectively, and ii) ANI TR and TT. The molecular structures of S-SWNTs and M-SWNTs covalently functionalized with PANI-ES and PANI-LS, respectively, are labeled in Figure S3 as PANI-LS/SWNTs and PANI-EB/SWNTs. In our opinion, these molecular structures can explain: i) the steric hindrance effects invoked as a result of the variation in the ratio between the absorbance of the IR bands situated in the spectral ranges 700–785 and 800–860 cm^-1^; ii) the change of the ratio between the relative intensities of the Raman lines situated at 1593–1596 and 1570 cm^-1^ in favor of the latter as a result of the covalent functionalization of the S-SWNT’s wall with PANI-salt; and iii) the absence of the Raman lines of the polymer after the interaction of the 1M NH_4_OH solution with the PANI-salt covalently functionalized M-SWNTs and S-SWNTs, respectively.


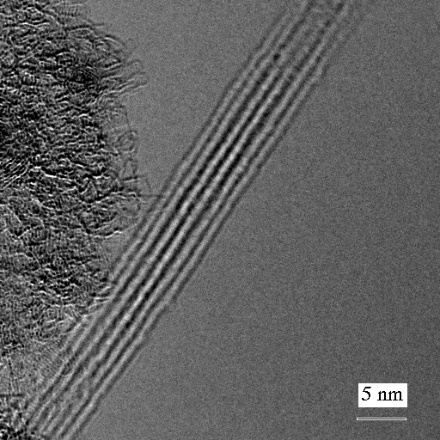

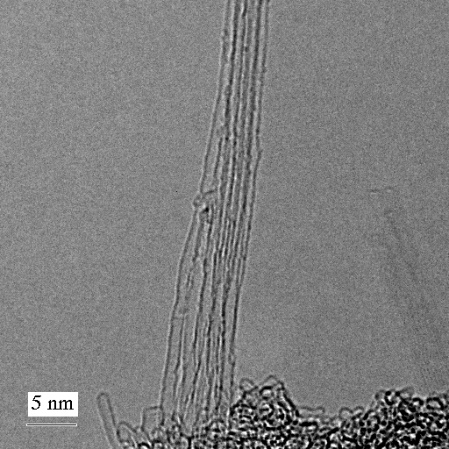


a b


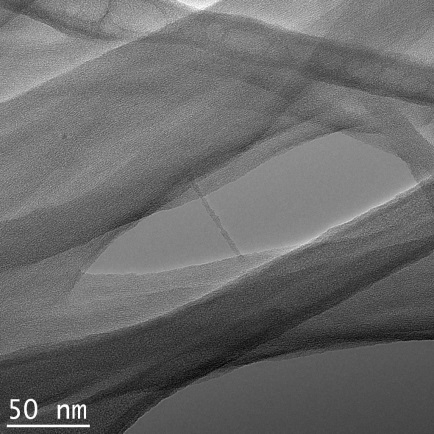

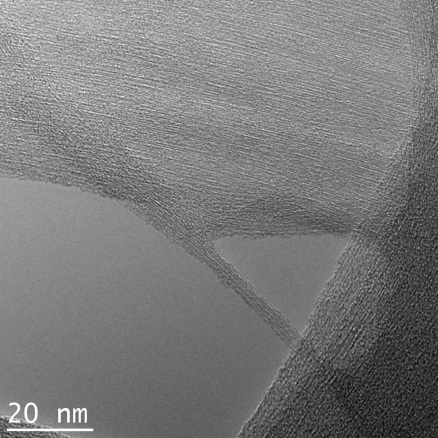
 c d

**Figure S11** HRTEM images of: **a**) S-SWNTs, **b**) M-SWNTs, **c**) S-SWNTs/ PANI doped with $\mathrm{Cl}^{-}$ ions and **d**) M-SWNTs/ PANI doped with $\mathrm{Cl}^{-}$ ions.


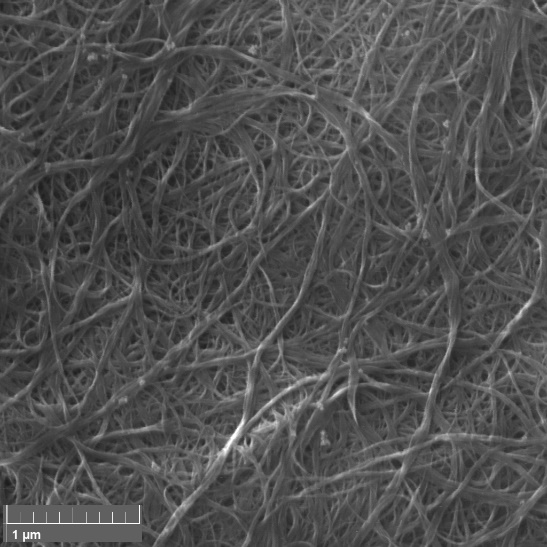

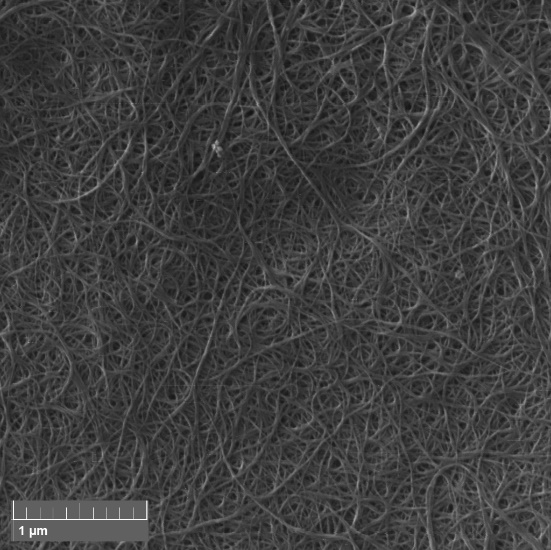
 a b


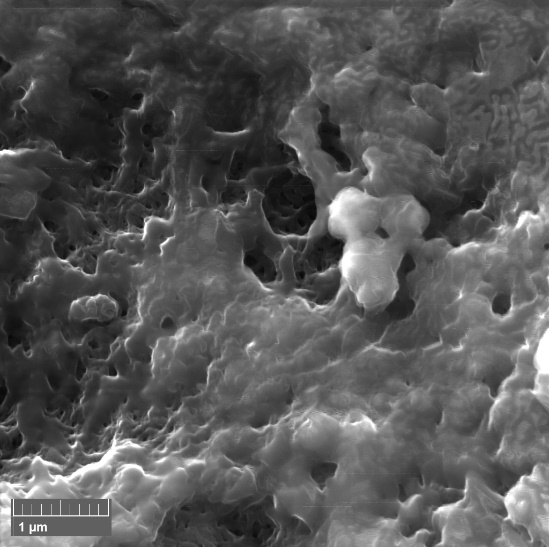

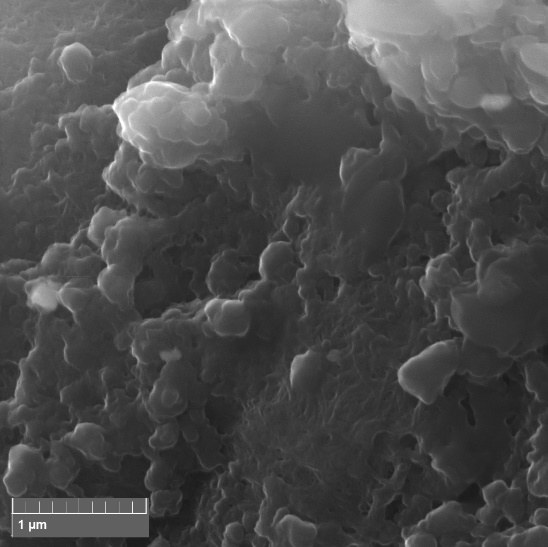


c d


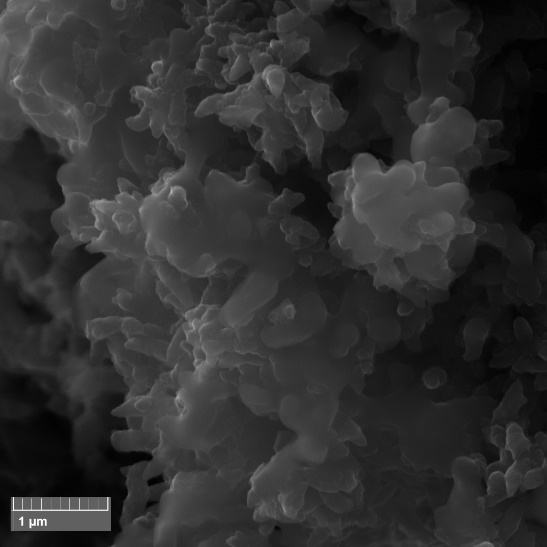

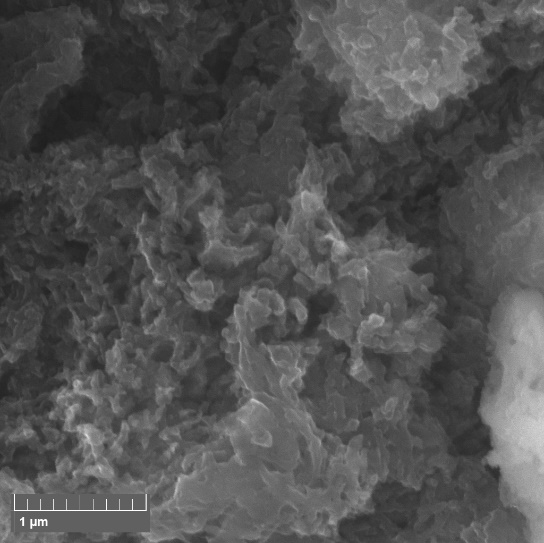


e f


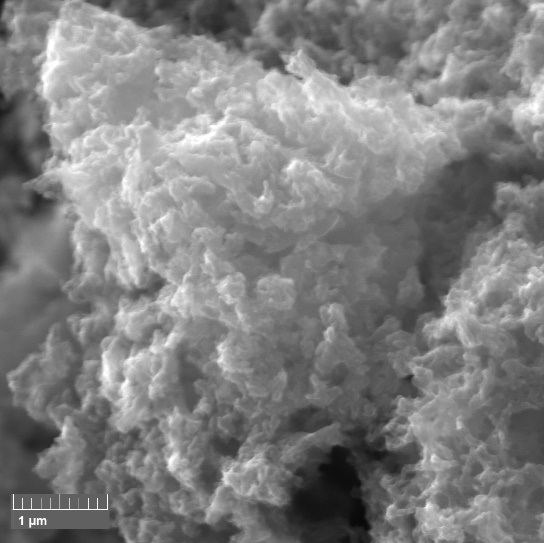

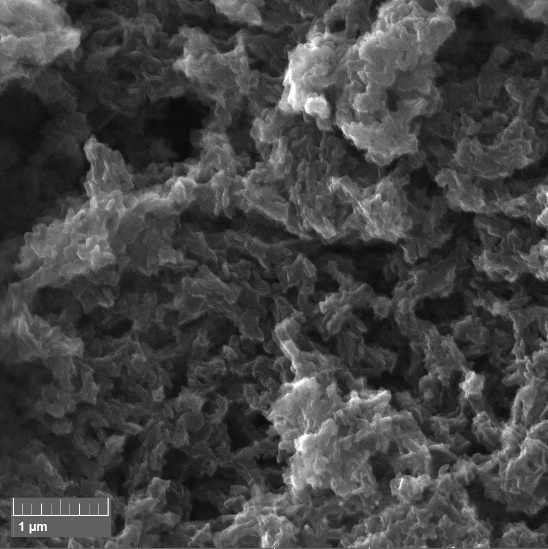


g f

**Figure S12** SEM images of the following samples: **a**) S-SWNTs, **b**) M-SWNTs, **c**) PANI doped with $\mathrm{HSO}_{4}^{-}$ ions, **d**) composite based on S-SWNTs and PANI doped with $\mathrm{HSO}_{4}^{-}$ ions, **e**) composite based on M-SWNTs and PANI doped with $\mathrm{HSO}_{4}^{-}$ ions, **f**) PANI doped with $\mathrm{Cl}^{-}$ ions, **g**) composite based on M-SWNTs and PANI doped with $\mathrm{Cl}^{-}$ ions and **h**) composite based on S-SWNTs and PANI doped with $\mathrm{Cl}^{-}$.

**References**

S1. Trchova, M., Stejskal, I. & Prokes, J. Infrared spectroscopic study of solid-state protonation and oxidation of polyaniline. *Synth. Met.* **101**, 840–841 (1999).

S2. Ping, Z., Nauer, G., Neugebauer, H., Theiner, J. & Neckel, A. Protonation and electrochemical redox doping processes of polyaniline in aqueous solutions: investigations using in-situ ftir-atr spectroscopy and a new doping system. *J. Chem. Soc.* *Faraday Transl.* **93**, 121–129 (1997).

S3. Martin, S., Salcedo, D., Molina, L. & Molina, M. Phase transformations of micron-sized H_2_SO_4_ / H_2_O particles studied by infrared spectroscopy. *J. Phys. Chem. B* **101**, 5307–5313 (1997).

S4. Baibarac, M., Baltog, I., Godon, C., Lefrant, S. & Chauvet, O. Covalent functionalization of single walled carbon nanotubes by aniline electrochemical polymerization. *Carbon* **42**, 3143–3152 (2004).

S5. Lapkowski, M., Berrada, S., Quillard, S., Louarn, G., Lefrant, S. & Pron, A. Electrochemical oxidation of polyaniline in nonaqueous electrolytes: ”in situ” Raman spectroscopic studies. *Macromolecules* **28**, 1233–1238 (1995).

S6. Cochet, M., Berrada, S., Louarn, G., Quillard, S., Buisson, J.P. & Lefrant, S. Theoretical and experimental vibrational study of emeraldine in salt form. part II. *J. Raman Spectrosc*. **31**, 1041–1049 (2000).

S7. Quillard, S., Louarn, G., Lefrant, S. & MacDiarmid, A. Vibrational analysis of polyaniline: A comparative study of leucoemeraldine, emeraldine, and pernigraniline bases. *Phys. Rev. B* **50**, 12496–12508 (1994).

S8. Boyer, M.I., Quillard, S., Rebourt, E., Louarn, G., Buisson, P., Monkman, A. & Lefrant, S. Vibrational analysis of polyaniline: a model compound approach. *J. Phys. Chem. B* **102**, 7382–7392 (1998).

S9. Garcia-Gallegos, J., Martin-Cullon, I., Conesa, J., Vega-Cantu, Y. & Rodriguez-Marcias, F. The effect of carbon nanofillers on the performance of electro-mechanical polyaniline-based composite actuators. *Nanotechnol*. **27**, 015501 (2016).
